# DRP1 depletion protects NK cells from hypoxia-induced dysfunction

**DOI:** 10.1080/13510002.2026.2626181

**Published:** 2026-02-18

**Authors:** Tias Verhezen, Astrid Van Den Eynde, Peter Verstraelen, Laura Gehrcken, Gabriele Palmiotto, Ho Wa Lau, Winnok H. De Vos, Sanne Van Der Heijden, Louize Brants, Jöran Melis, Jonas Van Audenaerde, Felicia Rodrigues Fortes, Maxim Le Compte, Geert Roeyen, Hans Prenen, Diana Campillo-Davo, Eva Lion, Rafael J. Argüello, Steven Van Laere, Filip Lardon, Christophe Deben, An Wouters, Evelien Smits, Jorrit De Waele

**Affiliations:** a Center for Oncological Research (CORE), Integrated Precision and Personalized Oncology Network (IPPON), University of Antwerp, Antwerpen, Belgium; b Laboratory of Cell Biology and Histology, University of Antwerp, Antwerpen, Belgium; c School of Medicine and Surgery, University of Bologna, Bologna, Italy; d Antwerp Centre for Advanced Microscopy, University of Antwerp, Antwerpen, Belgium; e µNEURO Research Centre of Excellence, University of Antwerp, Antwerpen, Belgium; f Department of Hepatobiliary Transplantation and Endocrine Surgery, Antwerp University Hospital, Edegem, Belgium; g Department of Oncology, Multidisciplinary Oncology Center Antwerp, Antwerp University Hospital, Edegem, Belgium; h Laboratory of Experimental Hematology (LEH), Vaccine and Infectious Disease Institute (VAXINFECTIO), University of Antwerp, Antwerpen, Belgium; i Aix Marseille University, CNRS, INSERM, CIML, Centre d'Immunologie de Marseille-Luminy, Marseille, France

**Keywords:** Natural killer cells, Tumor microenvironment, Hypoxia, Mitochondria, CRISPR-Cas9, ROS, Cancer, DRP1

## Abstract

**Objectives:**

The efficacy of cellular therapies has been disappointing in solid tumors. A major barrier that contributes to the low success rate, is hypoxia within the tumor microenvironment. In this study, we investigated the influence of hypoxia on natural killer (NK) cell function and to evaluated a strategy to restore their activity in hypoxia.

**Methods:**

Unarmed or CAR NK cells were placed in normoxia (21% O_2_) or hypoxia (1% O_2_) prior to experimental readouts. Mitochondrial content and morphology were assessed by confocal microscopy, membrane potential and reactive oxygen species (ROS) by flow cytometry, and global transcriptional changes by RNA sequencing. Cytotoxicity was evaluated against tumor cell lines and patient-derived cancer organoids, which were characterized by RNA sequencing. DRP1 function was inhibited pharmacologically or through CRISPR-Cas9-mediated knockout.

**Results:**

Hypoxia reduced NK cell mitochondrial content and membrane potential, while increasing mitochondrial ROS and inducing broad transcriptional changes in stress response pathways. Their cytotoxic activity was drastically impaired, which could not be prevented by CD70-CAR-IL-15 engineering. Pharmacological inhibition of DRP1 restored mitochondrial content and cytotoxic function. To confirm the role of DRP1, CRISPR-Cas9-mediated *DRP1* knockout (KO) NK cells preserved mitochondrial load and membrane potential under hypoxia, and DRP1^KO^ CAR NK cells retained cytotoxic activity under hypoxic conditions against cancer cell lines. Patient microtumor models with distinct transcriptomic profiles exhibited divergent responses to DRP1^WT^ and DRP1^KO^ CAR NK cells.

**Conclusion:**

These findings indicate that DRP1 inactivation supports NK cell function in hypoxia and metabolic engineering may enhance CAR-NK efficacy in solid tumors.

## Introduction

Cell and gene therapies are revolutionizing the way cancer is treated. Chimeric antigen receptor (CAR) T cell therapies have demonstrated the ability to induce durable remissions or even achieve cures in patients with advanced leukemia and lymphoma [1,2]. Despite these exciting results in hematological malignancies, the application of CAR-based therapies has been more challenging in solid tumors [3,4]. Up to 90 percent of adult cancers consist of solid tumors, many of which with poor prognosis, thus presenting a big need for new therapies [5]. Solid tumors have a complex tumor microenvironment (TME) that creates barriers for immune cell infiltration and function. This immune-suppressive niche is low in nutrients, highly acidic and hypoxic [6,7]. Accumulating data indicate that T cells and natural killer (NK) cells entering this hostile environment are rendered dysfunctional [8–12].

NK cells are cytotoxic cells from the innate immune system that can recognize and eradicate tumor cells swiftly without prior sensitization [13]. Their distinct advantages over T cells, including a superior safety profile and potential for allogeneic therapy, make NK cell treatments more economically and logistically feasible, broadening patient accessibility [14]. In addition, NK cells can eradicate tumor cells beyond their CAR construct due to their innate properties, which is a major benefit in the heterogeneous TME of solid tumors and the risk of antigen escape. The first-in-human clinical trial with CAR NK cells has shown promising results in CD19^+^ hematological malignancy [15]. Until now, efforts to enhance NK cell therapy efficacy in solid cancer have mainly focused on blocking inhibitory receptors, CAR engineering, NK cell engagers or cytokine stimulation. However, recognizing the profound impact of the TME on immunometabolism, recent approaches aim to combine immunotherapy with metabolic manipulation to bolster cellular function [16].

Hypoxia is a defining feature of most solid tumor types, arising from the rapid proliferation and altered metabolism of cancer cells, coupled with poorly organized and dysfunctional vasculature [6,17]. The imbalance between oxygen supply and demand creates steep oxygen gradients, with partial pressures dropping below 10 mmHg [18]. In normal tissues, the oxygen tension is in the range of 24-66 mmHg [19]. Hypoxia profoundly influences the behavior of cancer cells, leading to tumor progression, and contributes to resistance against chemotherapy, radiotherapy, and immunotherapy through a variety of mechanisms [17]. Clinically, hypoxia is associated with poor prognosis across a wide range of solid tumors [17]. Importantly, hypoxia also poses a major challenge for the efficacy of immune-based therapies, including adoptive cell therapies such as those using NK cells. The hypoxic TME can impair NK cell metabolism, survival, and effector functions, limiting their ability to eliminate tumor cells [8,12,20–22]. Understanding how hypoxia shapes NK cell biology is therefore critical for optimizing the design and application of NK cell-based immunotherapies in solid cancers.

Immune cell metabolism extends beyond ATP generation and biosynthesis, requiring coordinated metabolic pathways to support specialized effector functions. Mitochondria have emerged as key regulators of NK cell metabolism and fitness, and their architecture critically shapes NK cell responses in cancer and chronic infection [23]. Dysfunctional NK cells commonly display small, fragmented mitochondria, highlighting mitochondrial dynamics as a potential target for metabolic intervention [8,24]. The cytosolic GTPase dynamin-related protein 1 (DRP1) is the principal mediator of mitochondrial fission. Upon phosphorylation, DRP1 translocates to the outer mitochondrial membrane to drive fission events. Under cellular stress, dysregulated DRP1 activity promotes excessive fragmentation, mitochondrial dysfunction, elevated reactive oxygen species (ROS) production, and loss of cellular fitness [25]. These features suggest that modulating DRP1-dependent mitochondrial dynamics may restore NK cell function in hypoxic tumor microenvironments.

In this study, we investigated the influence of hypoxia on NK cells, including in the context of cytokine-armored fourth generation CAR NK cells. We confirmed that hypoxia impairs mitochondrial function and cytotoxic effector potential in NK cells. This dysfunction was also observed in CAR NK cells, despite autologous production of IL-15 to enhance persistence and metabolic fitness [26,27]. Building on the importance of mitochondrial fitness in NK cell cytotoxicity and the known role of DRP1 in hypoxia-induced mitochondrial fragmentation, we hypothesized that genetic ablation of DRP1 could preserve NK cell function under metabolic stress [8]. Both pharmacological inhibition and CRISPR-Cas9-mediated knockout (KO) of DRP1 in NK cells led to a better maintenance of mitochondrial content and cytotoxic potential in hypoxic conditions. This positions DRP1 depletion as an attractive avenue for improving NK cell therapy.

## Materials and methods

### Cell lines and culture conditions

NK-92 (ACC 488) and Raji (ACC 319) cell lines were purchased from the German Collection of Microorganisms and Cell Cultures (DSMZ). The PANC-1 (CRL-1469) cell line was purchased from the American Type Culture Collection (ATCC). HeLa and K562 cell lines were kindly provided by Dr. Eva González Suárez (Spanish National Cancer Research Centre, Spain) and Dr. Eva Lion (University of Antwerp, Belgium), respectively.

NK-92 cells were cultured in GlutaMAX alpha Minimum Essential Medium (α-MEM; 32561037, Life Technologies) supplemented with 12.5% Fetal Bovine Serum (FBS; 10270106, Life Technologies), 12.5% horse serum (16050122, Life Technologies), 2 mM L-glutamine (25030024, Life Technologies), 1% Penicillin/Streptomycin (P/S; 15140122, Life Technologies) and 150 U/mL recombinant IL-2 (11340027, ImmunoTools), as described before [28]. Raji cells were cultured in Roswell Park Memorial Institute Medium (52400025, Life Technologies) supplemented with 10% FBS, 2 mM L–glutamine, and 1% P/S. Panc-1 and HeLa cells were cultivated in Dulbecco Modified Eagle Medium (DMEM; 10938025, Life Technologies) supplemented with 10% FBS, 2 mM L-glutamine, and 1% P/S. Cell cultures were maintained in exponential growth at 37 °C in a humidified incubator with 5% CO₂. Regular testing using the MycoAlert Mycoplasma Detection Kit (LT07-318, Lonza) confirmed the absence of Mycoplasma contamination, and cell identity was validated through short tandem repeat profiling.

### Hypoxic environment

In this study, we used 1% O_2_ to model tumor hypoxia *in vitro*. This level is commonly used in cancer research to mimic the hypoxic microenvironment of solid tumors, where oxygen tensions often fall below 10 mmHg (∼1-1.5% O_2_) [12,18]. Furthermore, 1% O_2_ is sufficient to activate hypoxia-inducible pathways such as HIF-1α stabilization, providing a reliable model for studying hypoxic cellular responses [29]. Hypoxic conditions were reached by placing DRP1^WT^ and DRP1^KO^ NK-92 cells in H45 HEPA Hypoxystations (Don Whitley Scientific) at 1% O_2_ and 5% CO_2_ at 37 °C. Unless stated differently, cells were kept in hypoxia for 48 h before downstream experiments.

### Growth curve NK-92 cells

NK cell expansion was determined by calculating the fold increase in cell concentration. NK cells were initially seeded at a concentration of 0.333 × 10⁶ cells/mL in culture medium. After an incubation period of 3–4 days, the final cell concentration was measured using a TC20 Bio-Rad automated cell counter with Trypan Blue (15250061, Thermo Fisher Scientific) viability staining. This was repeated over the entire culture period.

### TCGA analysis

Gene expression data (RSEM format) for 32 different cancer types vouching for 10,071 individual cancer samples that are part of the pan-cancer analysis of whole genomes (PCAWG) series were downloaded from the cBioPortal for cancer genomics (https://www.cbioportal.org). Expression data were filtered to include genes with raw counts above 10 in at least 10% of the cases per cancer type, followed by library size scaling (count per million reads), log2 transformation and quantile normalization. Hypoxia scores were generated using gene set variation analysis (GSVA) based on a set of 15 hypoxia-related genes (*VEGFA*, *PGAM1*, *ENO1*, *LDHA*, *TPI1*, *P4HA1*, *MRPS17*, *ADM*, *NDRG1*, *TUBB6*, *ALDOA*, *MIF*, *SLC2A1*, *CDKN3*, *ACOT7*) identified by Buffa *et al.* (2010) in a meta-analysis across different cancer types [30]. Resulting hypoxia scores were compared between different tumor types.

### Generation of DRP1^KO^ NK-92 cells via CRISPR-Cas9

DRP1^KO^ NK cells were generated with CRISPR-Cas9 editing and validated by PCR amplification and flow cytometry staining as described previously [31]*.* Briefly*,* 1 × 10^6^ IL-2 pre-activated NK cells were nucleofected using a 4D Nucleofection (Lonza) in P3 solution (V4XP-3032, Lonza) with CRISPR-Cas9 (Alt-R S.p. Cas9 nuclease, 1081058, Integrated DNA Technologies) multi-guide ribonucleoprotein (RNP) complexes*.* The following sgRNA (synthetic guide RNA; Synthego) sequences were used to target the *DRP1* gene: (a) AUAUUCUGUUUUCAGAGCAG; (b) UUCCAGUACCUCUGGGAAGC; (c) GAAGAUAAACGGAAAACAAC. After 48 h, cells were harvested for genetic validation using PCR amplification. The following PCR primers were used to amplify the targeted *DRP1* sequence: forward, AGTCTCTGCACTAATTTTTCCTTT; and reverse, TGCATACTACTTCTCACAGGACTT. After validation on gene (PCR) and protein (flow cytometry) level, DRP1^KO^ NK cells were polyclonally expanded.

### Flow cytometry

All flow cytometry data was analyzed using FlowJo 10.9.0 (BD Life Sciences).

#### Co-culture with PKH67-stained K562 and Raji cells

Cytotoxic activity towards K562 and Raji cell lines was assessed by co-culturing NK cells with K562 or Raji cells in a 5:1 effector:target ratio in sterile FACS tubes (SIMPT416-6, VWR). Prior to start, tumor cells were labeled using PKH67 (MIDI67-1KT, Sigma-Aldrich) according to the manufacturer’s instructions. After 4 h, co-cultures were stained with 7-AAD (dilution 1:100; 420403, BD Biosciences) and Annexin V-PE (dilution 1:100; 556422, BD Biosciences). Tumor cell survival was measured on a CytoFLEX flow cytometer (Beckman Coulter). Supplementary Figure S1A and S1B show the gating strategy used to distinguish between live, apoptotic and dead K562 or Raji cells, respectively, in monoculture or coculture with (CAR) NK cells.

#### TMRM and MitoSOX staining

Mitochondrial membrane potential of NK cells was assessed by Tetramethylrhodamine, Methyl Ester, Perchlorate (TMRM; M20036, Thermo Fisher Scientific) staining and mitochondrial ROS levels were evaluated by MitoSOX mitochondrial superoxide indicators (M36008; Thermo Fisher Scientific). NK cells were harvested in U-bottom 96-well plates (650180, Greiner bio-one) and resolved in serum-free α-MEM medium. NK cells were incubated with 100 nM TMRM or 1 µM MitoSOX for 30 min at 37 °C, washed with FACS buffer, and stained with LIVE/DEAD™ Fixable Near-IR Dead Cell Stain (L10119, Thermo Fisher Scientific) for 15 min. TMRM and MitoSOX signals were measured using a NovoCyte Quanteon flow cytometer (Agilent Technologies), Mean Fluorescence Intensity (MFI) served as readout for TMRM and MitoSOX signals.

#### DRP1 staining

NK cells were harvested and washed with PBS-EDTA before staining with LIVE/DEAD™ Fixable Near-IR Dead Cell Stain (dilution 1:100; L34975, Thermo Fisher Scientific) for 15 min. Following incubation, cells were washed, fixed and permeabilized using eBioscience Foxp3 Fixation and Permeabilization Buffer Kit (00-5523-00, Thermo Fisher Scientific) according to the instructions of the manufacturer. To block non-specific binding, an intracellular blocking step was included with blocking buffer (permeabilization buffer with 10% human serum (1-LITER, Sigma-Aldrich)). Cells were then stained with Rabbit anti-Human Phospho-DRP1 (Ser616) Antibody (dilution 1:50; 3455, Cell Signaling Technology) in blocking buffer for 1 h, at 4 °C. This was followed by a washing step and a 30 min incubation with Goat anti-Rabbit IgG secondary antibody (Alexa Fluor™ 647; dilution 1:500; ab150079, Invitrogen) in permeabilization buffer. DRP1 signal (MFI) was measured using a NovoCyte Quanteon flow cytometer.

#### SCENITH™ assay

The SCENITH™ assay (GammaOmics, www.gammaomics.com) was performed as previously described by Argüello et al. [32]. Briefly, 0.25 × 10^6^ DRP1^WT^ or DRP1^KO^ NK cells were plated in a U-bottom, 96-well plate, treated with either control (DMSO), 2-Deoxy-D-Glucose (2-DG; 100 mM), oligomycin (Oligo; 1 μM), or a combination of the inhibitors at the same final concentrations, for 30 min at 37 °C. Following treatment with metabolic inhibitors, puromycin (Puro; 10 μg/mL) was added to the culture for the last 30 min. Cells were then washed with cold PBS and stained with LIVE/DEAD™ Fixable Aqua Dead Cell Stain Kit (L34957, Thermo Fisher Scientific) for 15 min. Subsequently, cells were washed, fixed and permeabilized using eBioscience Foxp3 Fixation and Permeabilization Buffer Kit. Intracellular block was performed with 10% human serum, followed by intracellular staining for puromycin using a monoclonal anti-puromycin antibody (1:50) in permeabilization buffer containing 10% human serum for 1 h at 4 °C. Puromycin signal was measured using a NovoCyte Quanteon flow cytometer. MFI was used to calculate SCENITH™ parameters based on the puromycin MFI for each condition, per gating strategy as depicted in Supplementary Figure S1C. The following formulas were applied:

- glucose dependence = 100 (Control – 2DG)/(Control – [2-DG + Oligo]);
- mitochondrial dependence = 100 (Control – Oligo)/(Control – [2-DG + Oligo]); and
- glycolytic capacity = 100 − mitochondrial dependence.

##### CD70 expression on cancer cells and CD70-targeting CAR expression on NK cells

Surface expression of the CD70-CAR-IL-15 constructs on NK cells was evaluated via flow cytometry by staining with a CD27-PE monoclonal antibody (mAb; dilution 1:100; 55584, Cell Signaling Technology) for 15 min. Expression of CD70 on tumor and cancer-associated fibroblast (CAF) cell lines was determined by staining with a CD70-PE mAb (dilution 1:50; CD70-PE, BD Biosciences) for 15 min. To exclude non-viable cells, the mAb stainings were combined with the LIVE/DEAD™ Fixable Near-IR Dead Cell Stain (dilution 1:100; L34975, Thermo Fisher Scientific). CD27 and CD70 expression were measured with a NovoCyte Quanteon flow cytometer.

### RNA sequencing

For RNA sequencing, DRP1^WT^ and DRP1^KO^ NK cells were harvested after 24 h of culturing in hypoxia or normoxia, washed with cold PBS, and stored as dry pellets at – 80 °C. NK cells were subjected to 24 h of hypoxia to ensure preservation of cell viability and transcriptional activity, which could be too low after 48 h of hypoxic culturing. Afterwards, RNA was extracted using RNeasy midi kit (74104, Qiagen). For removal of gDNA, RNAse-free DNAse treatment was performed. RNA concentration and purity were checked using the Qubit RNA BR Assay Kit on Qubit 4 Fluorometer (Q10210, Thermo Fisher Scientific) and NanoDrop ND-1000 (Thermo Fisher Scientific), respectively. Samples were frozen at – 80 °C and delivered to the Genomics Core Leuven (Leuven, Belgium) for transcriptome sequencing using QuantSeq 3’ mRNA-Seq V2 FWD Library Preparation Kit FWD (Lexogen) for Illumina on a Hiseq400 SR50 line (Illumina) with a minimum of 2 M reads per sample. Downstream analysis and plotting were performed using the Omics Playground tool (Big Omics Analytics) and RStudio (Posit PBC). These software tools were also used to analyze previously in-house generated RNA sequencing data on pancreatic ductal adenocarcinoma (PDAC) patient-derived organoids (GSE235548) [33].

### Confocal microscopy

#### Sample preparation

Following incubation under normoxic or hypoxic conditions, DRP1^WT^ and DRP1^KO^ NK cells were harvested, washed, and fixed in 2% paraformaldehyde (047377-9M, Thermo Scientific Chemicals) for 10 min. Subsequently, 1 × 10^5^ cells (at a concentration of 1 × 10^6^ cells/mL) were mounted onto glass slides using a CytoSpin 3 centrifuge (Thermo Shandon) at 1500 rpm for 5 min to ensure uniform cell distribution. To contain the sample area, a hydrophobic barrier was drawn around the mounted cells using a ReadyProbes™ Hydrophobic Barrier Pap Pen (R3777, Thermo Fisher Schientific). Cells were permeabilized with blocking buffer (PBS supplemented with 0.05% Thimerosal (71230, Fluka), 0.01% NaN3 (S2002, Acros), 0.1% bovine serum albumin (A1662-500 ml, Sigma) and 10% horse serum (16050130, Gibco)) containing 1% Triton X-100 (X100, Sigma) for 5 min. For mitochondrial staining, cells were incubated overnight with a primary mouse monoclonal anti-TOMM20 antibody (612278, BD Biosciences) diluted 1:250 in blocking buffer (4 °C). The following day, samples were washed with PBS and incubated with a Goat-anti-Mouse-AlexaFluor488-conjugated secondary antibody (1:500 dilution; A-11001, Invitrogen) along with CellMask (1:5000 dilution; H32721, Thermo Fisher Scientific) in blocking buffer for 2 h at room temperature. To visualize cell nuclei, cells were incubated with DAPI (5 µg/ml in PBS; D9542, Sigma) for 10 min at room temperature and washed with PBS.

#### Image quantification

High-resolution confocal Z-stacks (spacing: 0.1 µm) were acquired on a Nikon CSU-W1-SoRa spinning disk confocal with a 100X silicone immersion objective (NA 1.35), using standard filters for blue (DAPI), green (AlexaFluor488) and far red (CellMask). Three-dimensional image analysis was carried out in Arivis software (Zeiss). Briefly, the DAPI channel was segmented with a blob finder to delineate nuclei, which were then used as seeds to segment the cell based on the CellMask channel. Within the cytoplasm, i.e. cell minus nucleus, mitochondria were detected in the TOMM20 channel. Mitochondrial content was calculated as the total volume of all segmented mitochondria divided by the volume of the cytoplasm. Numerical data was plotted and analyzed in RStudio. One data point represents one cell, originating from 4 independent experiments.

### Generation of CD70-targeting IL15-expressing CAR NK cells

CD70-CAR-IL-15 constructs were designed and created as previously described [28]. *In vitro* transcription of CD70-CAR-IL-15-encoding messenger RNA (mRNA) was performed using the mMESSAGE mMACHINE T7 transcription kit (AM1344, Life Technologies) following manufacturer’s instructions. DRP1^WT^ or DRP1^KO^ NK-92 cells were electroporated using a 4D Nucleofector device (Lonza) in the presence of 4 μg CD70-CAR-IL-15-encoding mRNA per 1 million DRP1^WT^ or DRP1^KO^ NK cells.

### xCELLigence co-culture

Longitudinal cytotoxic activity of CD70-CAR-IL-15 DRP1^WT^ and DRP1^KO^ NK cells towards CD70^+^ cell lines (HeLa and Panc-1) was analyzed using the xCELLigence Real-Time Cell Analysis (RTCA; Agilent) that records cell viability and growth by impedance measurements. Seeding density was optimized for each cell line to ensure continuous growth until the end of the assay. Target cells were seeded in gold-coated 16-well plates (380601310, Agilent) and background impedance of the plates was measured before seeding of the target cells. After a 24 h incubation to allow proper adhesion to the plates, cell lines were treated with CD70-CAR-IL-15 DRP1^WT^ and DRP1^KO^ NK cells in a 5:1 effector:target ratio (based on the amount of target cells seeded at day 0), or left untreated. The impedance signal was monitored by automated measurements every 15 min starting from cell seeding and ending 24 h after treatment. Measurement of the impedance was expressed as Cell Index and normalized to 1 after starting the co-culture.

### Microtumor co-culture

#### Organoid preparation

Longitudinal cytotoxic activity of DRP1^WT^ and DRP1^KO^ CD70-CAR-IL-15 NK-92 cells was evaluated using 3D patient-derived PDAC organoids, combined with RLT-PSC CD70^+^ CAF cells to form microtumors. PDAC organoids from three patients (Patient 1-3) were available through our in-house organoid bank, generated in accordance to the Declaration of Helsinki and approved by UZA Ethical Committee (ref. 14/47/480) [33,34]. In short, organoids were digested into single-cell suspension using TrypLE (12604021, Life Technologies) and plated in 100% Cultrex (3700-100-01, Bio-Techne Ltd) drops on flat-bottom culture plates (10369081, Greiner bio-one). The drops were covered with Advanced DMEM/F12 medium (12634010, Life Technologies) supplemented with 1% GlutaMAX (35050061, Life Technologies), 1% HEPES (15630080, Life Technologies), 1% P/S, 4% Noggin-Fc Fusion Protein (N002-100 ml, ImmunoPrecise Antibodies), 4% R-Spondin-Fc Fusion Protein (R001-100 ml, ImmunoPrecise Antibodies), 1 × B27 supplement (11530536, Life Technologies), 1.25 mM N-Acetylcysteine (A9165, Sigma-Aldrich), 10 mM Nicotinamine (72340, Sigma-Aldrich), 500 nM A83 (2939, Bio-Techne), and 5 nM Wnt Surrogate-Fc Fusion Protein (PHG0402, ImmunoPrecise Antibodies). For downstream microtumor generation, organoids were mixed with green fluorescent-transduced RLT-PSC cells, seeded in Advanced DMEM/F12 medium containing 3% Cultrex, supplemented with 1% GlutaMAX, 1% HEPES, and 1% P/S in 384-well ultra-low attachment microplates (4588, Corning), and incubated at 37°C for three days to allow for microtumor assembly.

#### Image quantification

Following treatment with CD70-CAR-IL-15 DRP1^WT^ or DRP1^KO^ NK cells, microtumors were longitudinally monitored every 2 h for 36 h using a Celldiscoverer 7 with LSM 900 (Zeiss). Brightfield and confocal images were analyzed using Arivis analysis software and growth rate of RLT-PSC cells in the microtumors was determined by normalizing the fluorescent green signal to the first measurement (T0) or the control (untreated) at endpoint (36 h). Briefly, we used a machine learning based classifier to segment the organoids in the brightfield (and green signal) channel (ROI-set-1). Separately, we used an intensity threshold, set to a value derived by the intensity of the green signal in the final timepoint of the control (no NK) well (ROI-set-2). We chose this value to correct for the fact that the organoids over the course of the experiment time consolidated and became denser, diminishing the total green signal. We then summed up the area of both segmentation results (ROI-set-1 U ROI-set-2) to obtain the final masks.

### Statistics

Experiments were conducted with a minimum of three biological replicates. ‘n’ refers to independent biological replicates in the figure legends. In the microtumor assay, each experimental condition was assessed using three technical replicates per patient sample (three biological replicates with three patient samples). Statistical analyses were performed using GraphPad Prism v10.2.2 (GraphPad Software Inc.), JMP Pro v17.0.0 (SAS Institute Inc.), and RStudio version R 4.4.2. To compare two groups, either parametric paired t-tests or non-parametric Wilcoxon signed-rank tests were used, depending on data distribution and sample size. For comparisons involving more than two groups, one-way ANOVA or Linear Mixed Models were applied. Tukey’s post hoc correction was used for all pairwise comparisons to adjust for multiple testing. A *p*-value < 0.05 was considered statistically significant.

## Results

### Hypoxia impairs NK cell cytotoxic effector potential and is associated with mitochondrial dysfunction

**Figure 1.**
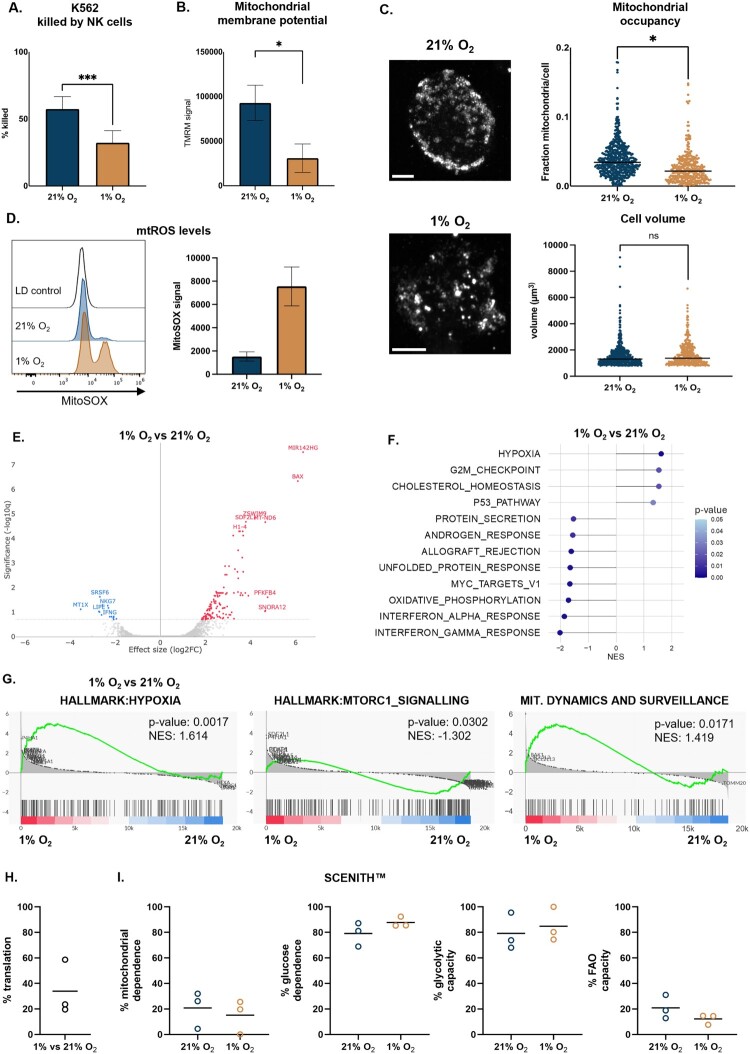
**Hypoxia affects NK cell cytotoxic effector potential, gene expression, and metabolism**. A. Cytotoxic activity (% K562 cells killed) of NK cells cultured in normoxia (21% O₂) or hypoxia (1% O₂), measured by flow cytometry (n = 6); B. Mitochondrial membrane potential measured by TMRM staining in NK cells after 48 h in normoxia or hypoxia, analyzed by flow cytometry (n = 3); C. Representative confocal microscopy images of NK cells showing mitochondria (TOMM20) after 48 h in normoxic or hypoxic conditions. Each point represents one cell (n = 4). Scale bar = 5 µm; D. mtROS levels after normoxic/hypoxic culturing as determined by mitoSOX staining (n = 3); Data in panels E-G are based on bulk RNA-seq (n = 3): E. Volcano plot of differentially expressed genes identified between the hypoxia treated and normoxia control cells; F. Hallmark gene set enrichment analysis (GSEA) showing the top up- and downregulated pathways in hypoxia, visualized as a lollipop plot; G. GSEA enrichment plots showing increased expression of hypoxia-related genes and mitochondrial dynamics and surveillance genes, with reduced mTORC1 signaling in hypoxic NK cells; H-I. SCENITH™-based analysis of NK cell metabolic activity under normoxia and hypoxia, showing corresponding protein translation level (H) and metabolic phenotypes (I) (n = 3). *p < 0.05; ***p < 0.001; ns = not significant. FAO: Fatty Acid Oxidation.

Improving NK cell performance in the hypoxic TME requires a clear understanding of how low oxygen alters their metabolism and cytotoxicity. We therefore modeled TME-like oxygen levels by culturing NK cells at 1% O₂ for 48 h, with target cells maintained at 21% O₂ serving as normoxic controls. Hypoxia significantly impaired NK cell cytotoxicity towards K562 target cells, confirming that low oxygen compromises their effector function (Figure 1A).

To identify potential metabolic contributors to this dysfunction, we evaluated mitochondrial content in NK cells exposed to hypoxia. The total mitochondrial content (expressed as volume occupancy) was not affected after 24 h, but a significant reduction was observed at 48 h, while cell volume was unchanged (Figure 1C, S2A). Moreover, mitochondrial membrane potential, a proxy for mitochondrial activity, was significantly lower in NK cells cultured under hypoxia (Figure 1B), while mitochondrial ROS (mtROS) levels were strongly elevated (Figure 1D). Together, these observations are indicative of mitochondrial stress due to hypoxic culturing.

To further understand how hypoxia alters NK cell metabolism, we performed RNA sequencing on NK cells cultured under normoxic and hypoxic conditions. Differential gene expression analysis revealed a distinct transcriptional profile in hypoxic NK cells, with upregulation of genes involved in stress and metabolic adaptation (Figure 1E). Among the most significantly upregulated genes in hypoxia, we identified MIR142HG, a non-coding RNA molecule implicated in NK cell immune regulation and metabolic adaptation [35]. Additionally, the pro-apoptotic gene *BAX* was significantly upregulated, suggesting potential stress-induced apoptotic priming. As expected, hypoxia-exposed NK cells exhibited significant enrichment of hypoxia hallmark genes (Figure 1F). Conversely, genes associated with mTORC1 signaling, interferon responses, and OXPHOS were significantly downregulated, while mitochondrial dynamics and surveillance genes were upregulated (Figure 1F, G). mTORC1 signaling plays a central role in regulating NK cell metabolism and effector function, while interferon responses are essential for immune surveillance and cytotoxicity [36]. The downregulation of OXPHOS-related genes is consistent with the reduced mitochondrial load and membrane potential observed in hypoxic NK cells (Figure 1B, C, F). The upregulated ‘mitochondrial dynamics and surveillance’ transcriptional signature highlights a mitochondrial stress response in hypoxic NK cells, characterized by increased expression of genes regulating fission (*e.g. DNM1L*, *FIS1*, *MFF*), fusion (*e.g. MFN1*, *MFN2*, *OPA1*), apoptosis (*BAX, BAK1, BNIP3*, caspases), and mitophagy (*PINK1, PRKN, BNIP3*) [37]. In addition, hypoxic NK cells show a trend towards reduced antioxidant activity, which align with the increased mtROS observed (Figure 1D, S2B). Altogether, these findings suggest that hypoxia drives a shift toward enhanced mitochondrial remodeling and quality control, potentially priming NK cells for functional exhaustion or apoptotic susceptibility.

To functionally validate these shifts in metabolic programming, we performed SCENITH™, a flow cytometry-based assay that quantifies protein synthesis in response to metabolic pathway inhibition, based on puromycin incorporation [32]. Hypoxia caused an approximately 4.5-fold reduction in puromycin MFI in control-treated cells, indicating a marked suppression of global protein translation, and thus ATP production, which is expected in a hypoxic environment [38] (Figure 1H, S2C). In addition, though not statistically significant, glycolytic capacity and glucose dependency were increased in hypoxia, while mitochondrial dependence was decreased, which is consistent with a reduced reliance on OXPHOS, as indicated by RNA-seq (Figure 1F, I, S2D). This reflects the expected glycolytic adaptation in hypoxia at the expense of mitochondrial respiration [39]. Together, these observations indicate that hypoxia places significant metabolic and survival stress on NK cells, leading to mitochondrial dysfunction and functional impairment.

### Cytokine-armed CAR engineering fails to restore NK cell function in hypoxia

While NK cells possess intrinsic antitumor activity, cytokine-armored CAR engineering is often required to maximize anti-cancer efficacy by enhancing target specificity and persistence, especially in solid tumors [40]. We previously showed that co-expression of IL-15 with a CD70-directed CAR enables robust anti-tumor activity *in vitro* and *in vivo* against hematological and solid tumor models [28]. Since it is known that IL-15 supports NK-cell metabolic fitness, we investigated whether these CAR-engineered cells could withstand hypoxic stress [24,36].

**Figure 2.**
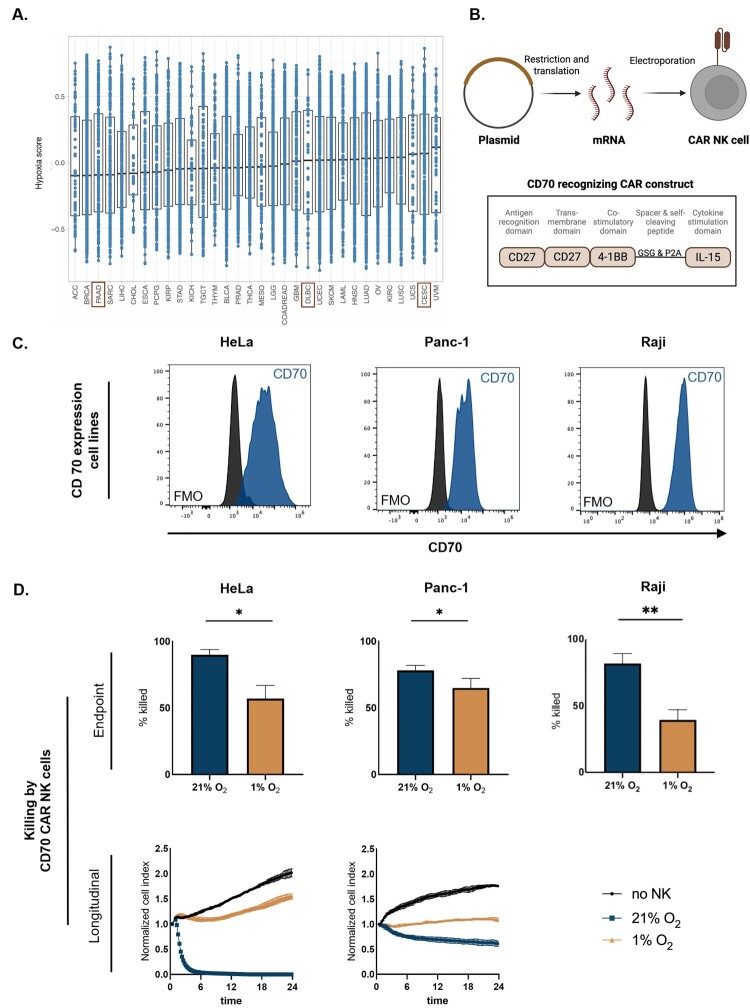
**CD70-CAR-IL-15 NK cells are rendered dysfunctional in hypoxia**. A. Hypoxia scores across cancer types in TCGA; hypoxia scores were calculated based on the hypoxia gene signature by Buffa *et al.* (2010) applied to bulk RNA-seq data from The Cancer Genome Atlas (TCGA) across multiple cancer types [30]. Each dot represents one patient. Cancer types are labeled using standard TCGA abbreviations (see Supplementary Table 1 for full names). B. Schematic representation of CAR mRNA synthesis and electroporation into NK cells, based on the protocol described by Van den Eynde *et al.* [28]; C. CD70 surface expression on HeLa, Panc-1, and Raji target cell lines, as determined by flow cytometry, blue histograms represent CD70 staining and black histograms show fluorescence minus one (FMO) controls; D. Cytotoxicity of CD70-CAR-IL-15 NK cells against CD70^+^ targets under normoxic (21% O₂) and hypoxic (1% O₂) conditions. For HeLa and Panc-1, longitudinal killing was measured by xCELLigence (normalized cell index over 24 h; n = 6), while Raji killing was assessed by flow cytometry after 4 h. Bar charts show endpoint % target cell killing and curves show real-time cytolytic activity (n = 6). *p < 0.05; **p < 0.01.

To explore the interaction of CAR NK cells with tumors that can exhibit hypoxia, we first assessed the degree of hypoxia across tumor types using The Cancer Genome Atlas (TCGA) dataset. We applied a validated hypoxia gene signature to bulk RNA-seq data across multiple cancer types (Figure 2A and Table 1 in supplementary). Many tumor types exhibited elevated hypoxia scores, with several well-known hypoxic malignancies (e.g. glioblastoma, head and neck squamous cell carcinoma, sarcoma) scoring highly. Interestingly, pancreatic adenocarcinoma (i.e. PAAD), despite being characterized by profound hypoxia at the microenvironmental level [41], displayed only intermediate hypoxia scores in the TCGA dataset. It is to note that each cancer type features a substantial amount of tumor specimens that score highly on the hypoxia signature. Based on both the TCGA data and the established hypoxic microenvironments of selected tumor entities, we selected a representative panel of tumor cell lines for *in vitro* modeling. These included HeLa (cervical carcinoma; CESC), PANC-1 (pancreatic ductal adenocarcinoma; PAAD), and Raji (Burkitt lymphoma; approximating DLBC). This panel enabled us to evaluate NK cell interactions across both solid tumors and hematologic malignancies, incorporating tumor types with variable baseline hypoxia profiles.

We generated CD70-CAR-IL-15 NK cells (Figure 2B; CAR expression in Figure S3) and confirmed high CD70 surface expression on all three hypoxic target cancer cell lines (Panc-1, HeLa, and Raji) (Figure 2C). CAR NK cells were pre-conditioned for 48 h in either normoxia or hypoxia prior to co-culture. Hypoxia significantly reduced CD70-CAR-IL-15 NK cell-mediated killing across all targets (Figure 2D). The magnitude of suppression was most pronounced in Raji and HeLa, while the effect on Panc-1 cells was more modest. These results demonstrate that, even with IL-15 support, hypoxia imposes a suppressive effect on antigen-specific CAR NK cell function in hematological and solid tumor cell lines.

### DRP1 modulation alleviates the adverse effects of hypoxia

**Figure 3.**
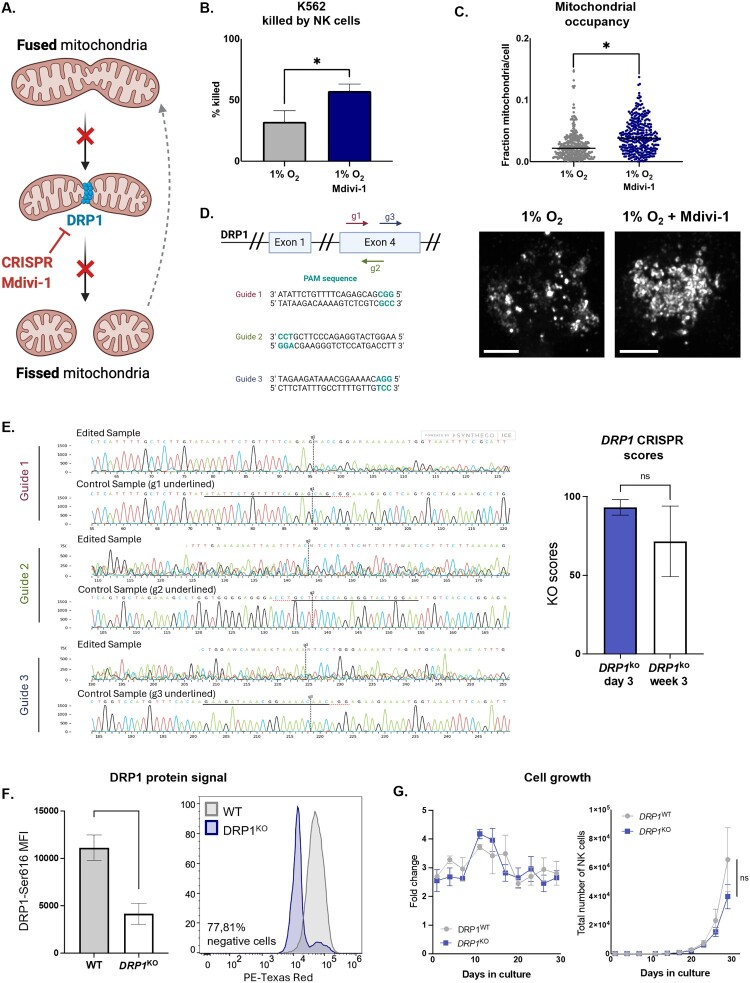
**CRISPR-Cas9-mediated deletion of *DRP1* in NK cells**. A. Schematic representation of DRP1-mediated mitochondrial fission and its inhibition by Mdivi-1 or CRISPR-Cas9-mediated knockout (KO); B. Cytotoxic activity (% K562 cells killed) of NK cells cultured under hypoxia (1% O₂) with or without Mdivi-1 treatment (n = 6); C. Mitochondrial occupancy in hypoxic NK cells with or without Mdivi-1, as measured by confocal microscopy (n = 4). Representative images shown. Scale bar = 5 µm; D. Schematic of the *DRP1* gene with target sites for the three guide RNAs used for CRISPR editing; E. Representative Sanger sequencing chromatograms (left) for each guide RNA and corresponding KO efficiency scores (right) at day 3 and week 3 post-editing, analyzed with Synthego’s ICE tool; F. Flow cytometric quantification of DRP1 protein expression in DRP1^KO^ vs DRP1^WT^ NK cells (n = 3); G. Growth curves of DRP1^WT^ vs DRP1^KO^ NK cells (n = 3). *p < 0.05; **p < 0.01; ns = not significant.

Hypoxia impaired mitochondrial content and function, while enhancing mitochondrial dynamics, and CD70-CAR-IL-15 engineering failed to restore cytotoxicity. DRP1 has been implicated in hypoxia-induced mitochondrial fragmentation, which leads to mitochondrial crippling and redox imbalance, resulting in impaired NK cell function in solid tumors [8]. Therefore, we next investigated whether inhibiting the fission factor DRP1 could mitigate NK cell dysfunction. To test this, we used the pharmacological DRP1 inhibitor Mitochondrial Division Inhibitor 1 (Mdivi-1) (Figure 3A). Indeed, blocking this fission factor through Mdivi-1 treatment restored K562 killing under hypoxia and prevented the loss of mitochondrial content (Figure 3B, C). However, the specificity of this small molecule has been questioned, as it may affect cellular metabolic pathways beyond mitochondrial fragmentation [42]. Moreover, its clinical applicability remains limited due to the lack of safety data.

To overcome these limitations, we generated a stable KO of the *DRP1* gene using CRISPR-Cas9 technology. Engineering a cell product with targeted gene knockout avoids systemic exposure to Mdivi-1. We achieved high and durable KO efficiency by using pre-formed Cas9 protein and a multi-guide RNA strategy targeting exon 4, as confirmed by Sanger sequencing (Figure 3D, E and Figure S4). Consequently, DRP1^KO^ NK cells showed markedly reduced phospho-DRP1 levels, confirmed by flow cytometry, demonstrating efficient knockout at the protein level. Given the central role of mitochondrial dynamics in cellular health and cell division, we assessed whether DRP1 ablation affected NK cell proliferation over time. Importantly, DRP1-ablation maintained proliferation in NK cells at comparable rate to wild-type controls (Figure 3F, G). Hence, while DRP1 is implicated in hypoxia-induced mitochondrial and functional NK cell impairment [8], its loss does not compromise cell viability or proliferative capacity, setting the stage for functional assessment.

### DRP1^KO^ NK cells display features of improved mitochondrial resilience under hypoxia

**Figure 4.**
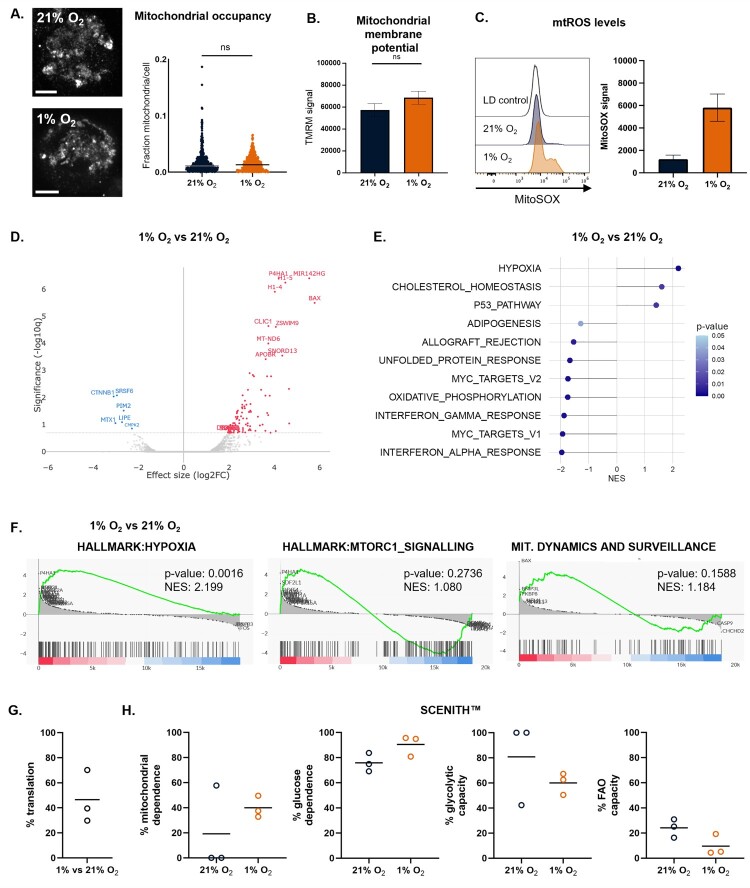
**Mitochondrial, transcriptomic, and metabolic profiling of DRP1^KO^ NK cells under hypoxia**. A. Mitochondrial occupancy in DRP1^KO^ NK cells cultured in normoxic (21% O₂) or hypoxic (1% O₂) conditions, assessed by 3D confocal imaging, with each point representing a single cell. Representative images shown. Scale bar = 5 µm; B. Mitochondrial membrane potential measured by TMRM staining in DRP1^KO^ NK cells under normoxia or hypoxia, analyzed by flow cytometry (n = 3); C. mtROS levels after normoxic/hypoxic culturing as determined by mitoSOX staining (n = 3); Data in panels D-F are based on bulk RNA-seq (n = 3): D. Volcano plot showing differentially expressed genes between DRP1^KO^ NK cells cultured in hypoxia (1% O₂) versus normoxia (21% O₂); significantly upregulated and downregulated genes are highlighted; E. Hallmark gene set enrichment analysis displaying the top up- and downregulated pathways in hypoxic versus normoxic DRP1^KO^ NK cells; F. Gene set enrichment analysis (GSEA) plots demonstrating increased expression of the hypoxia gene signature, no significant reduction in mTORC1 signaling and no significant upregulation of mitochondrial dynamics genes in hypoxic DRP1^KO^ NK cells; G-H. SCENITH™-based analysis of metabolic activity in DRP1^KO^ NK cells under normoxia and hypoxia, showing corresponding protein translation level (G) and metabolic phenotypes (H) (n = 3).

Firstly, we assessed mitochondrial occupancy of DRP1^KO^ cells in hypoxia. DRP1^KO^ NK cells maintained mitochondrial integrity under hypoxic conditions, with mitochondrial occupancy preserved at levels comparable to normoxia (Figure 4A, S5A). Mitochondrial membrane potential remained stable under hypoxia (Figure 4B), while mtROS levels were still elevated in hypoxia (Figure 4C).

Next, we investigated the transcriptional and metabolic profiles of DRP1^KO^ cells in hypoxic and normoxic conditions. The gene expression profile and top up- and downregulated hallmark pathways were similar to the DRP1^WT^ cells in normoxic vs hypoxic conditions (Figure 4D, E), with MIR142HG and BAX remaining the most strongly upregulated genes in hypoxic DRP1^KO^ NK cells. However, notable exceptions were the mTORC1 hallmark and the mitochondrial dynamics signature. While the mTORC1 pathway was downregulated in hypoxic DRP1^WT^ NK cells (Figure 1F), it appeared unchanged in DRP1^KO^ cells under the same conditions (Figure 4F). Given mTORC1s role in NK cell effector function [36], this might be indicative of preserved killing capacity. In addition, while the mitochondrial dynamics signature was significantly upregulated in DRP1^WT^ NK cells (Figure 1G), this was not the case in DRP1^KO^ NK cells (Figure 4F), consistent with an unchanged mitochondrial load. A gene signature for antioxidant activity was trending towards downregulation in hypoxia (Figure S5B), aligning to the increased mtROS levels and similar to DRP1^WT^ NK cells (Figure 4C, S2B).

To determine whether DRP1 ablation alters the metabolic response of NK cells to hypoxia, we performed SCENITH™ analysis on DRP1^KO^ cells cultured under normoxic and hypoxic conditions. Similar to wild-type cells, DRP1^KO^ NK cells displayed reduced translation and ATP production in hypoxia (Figure 4G, S5C). Interestingly, despite the downregulation of OXPHOS-associated genes in the RNA-seq data, SCENITH™ revealed increased mitochondrial dependence in hypoxia, consistent with the preserved mitochondrial membrane potential and mitochondrial content observed in DRP1^KO^ cells (Figure 4H). Overall, DRP1^KO^ NK cells maintained mitochondrial content, membrane potential, and mTORC1 signaling under hypoxic conditions, supporting their potential to resist hypoxia-induced dysfunction.

### DRP1^KO^ CAR NK cells retain their cytotoxic effector potential in hypoxia

**Figure 5.**
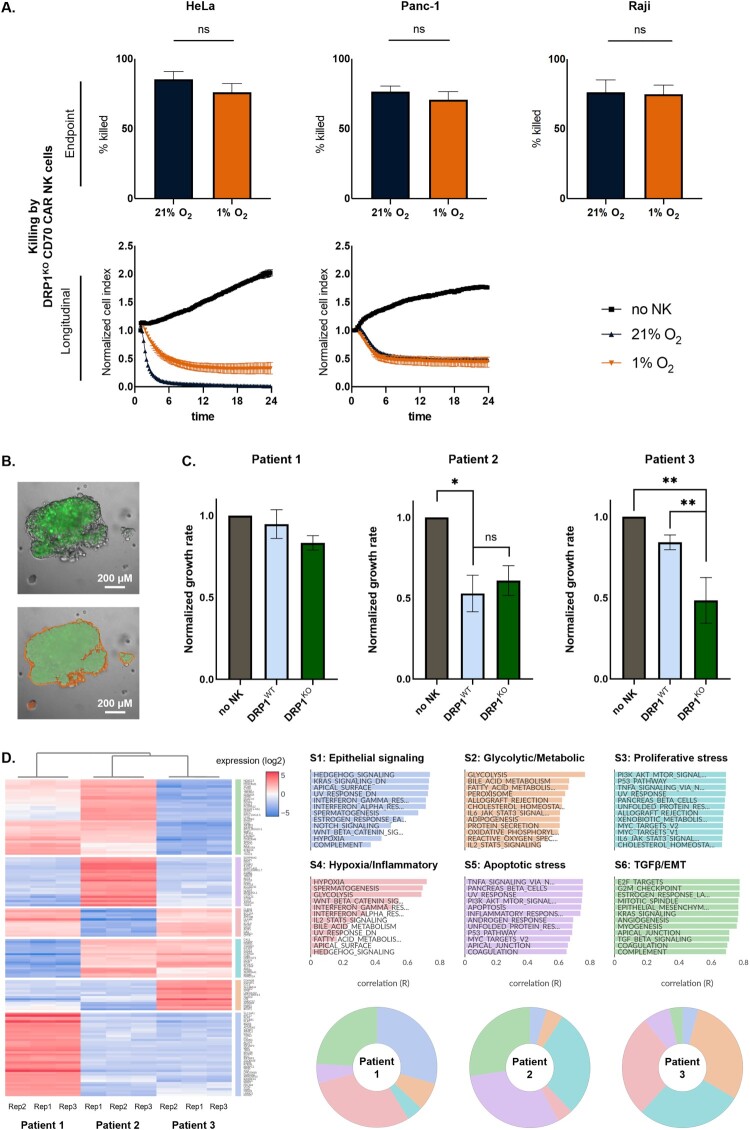
**DRP1^KO^ cytotoxic effector potential in cell lines and microtumors**. A. Cytotoxic activity of DRP1^KO^ CD70-CAR-IL-15 NK cells against HeLa, Panc-1, and Raji target cells cultured in normoxic (21% O_2_) or hypoxic (1% O_2_) conditions, cytotoxicity was measured by xCELLigence (HeLa, Panc-1; n = 7) and flow cytometry (Raji; n = 7), upper panels show endpoint % killing and lower panels show longitudinal normalized cell index over 24 h; B. Representative confocal images of patient-derived pancreatic ductal adenocarcinoma (PDAC) organoid-CAF microtumors Top: fluorescent signal from CAFs. Bottom: corresponding segmentation mask used for quantification; C. Quantification of normalized green CAF signal 36 h post CAR NK cell treatment, relative to untreated control (n = 3 technical replicates). *p < 0.05; **p < 0.01; ns = not significant; D. Unsupervised clustering of bulk RNA-sequencing of three PDAC patient-derived organoids revealed distinct transcriptional patterns per patient across six transcriptional modules, each representing a dominant biological program.

To assess whether the preserved mitochondrial integrity of DRP1^KO^ NK cells translate into improved effector function, we engineered these cells to express the CD70-CAR-IL-15 construct (Figure S6A) and evaluated their cytotoxic potential under hypoxic conditions. DRP1^KO^ CAR NK cells maintained robust cytotoxic effector function across all three tested tumor cell lines (HeLa, Panc-1 and Raji), showing no impairment under hypoxia compared to normoxia (Figure 5A).

To further evaluate their functionality in a more physiologically relevant context, we established 3D microtumors composed of PDAC patient-derived organoids with CD70^+^ CAFs (Figure 5B). This model better recapitulates the cellular complexity of the TME and allows assessment of patient-specific responses. In one out of three tested patient samples (Patient 3), DRP1^KO^ CAR NK cells significantly reduced green CAF signal (mean normalized growth rate = 0.4840 ± 0.1993) compared to DRP1^WT^ CAR NK cells (mean = 0.8420 ± 0.09068) and the no-NK control (normalized to 1). In microtumors from the other two patients (Patients 1 and 2), no significant differences were observed between responses to DRP1^WT^ and DRP1^KO^ CAR NK cells, with Patient 1 and Patient 2 showing resistance and sensitivity, respectively (Figure 5C). To investigate why microtumors from different patients responded differently to CAR NK cells, we performed RNA sequencing on patient-derived organoids to identify distinct transcriptional tumor programs associated with the CAR NK cell activity (Figure 5D). Patient 1 organoids showed enrichment in epithelial signaling (S1), hypoxia and inflammatory stress (S4), and TGF-β signaling and epithelial-to-mesenchymal (EMT) transition (S6), reflecting a TGF-β^high^, EMT-skewed phenotype, which may underlie the complete resistance both DRP1^WT^ and DRP1^KO^ CAR NK cells. Patient 2 organoids displayed proliferative (S3) and apoptotic stress (S5) signatures, along with TGF-β/EMT (S6), reflecting a stressed tumor state that aligned with robust basal NK cell activity. Patient 3 organoids exhibited strong glycolysis and metabolism (S2), proliferative stress (S3) and hypoxia and inflammation (S4) programs, defining a hypoxia-adapted, glycolytic phenotype. While these microtumors were resistant to DRP1^WT^ NK cells, they were efficiently targeted by DRP1^KO^ NK cells, underscoring their resilience to hypoxic stress. These findings indicate that the transcriptional state of patient-derived organoids define the microtumor resistance patterns to CAR NK cells, highlighting the importance of patient variability when evaluating NK cell therapies. In summary, DRP1 ablation can enhance NK cell anti-tumor function in a context-dependent manner, supporting its potential as a metabolic intervention strategy.

## Discussion

Hypoxia is a hallmark of solid tumors and is known to compromise the function of immune cells, including NK cells [6,8,12]. Consistent with earlier findings, our results show that exposure to low oxygen levels leads to reduced NK cell cytotoxicity [8,12]. This impairment was also observed in NK cells engineered with a CD70-targeting CAR and autologous IL-15 secretion. While CAR-based strategies and cytokine support enhance NK cell function in general, they proved insufficient to fully counteract hypoxia-induced dysfunction. Consistently, Kennedy *et al.* reported that exogeneous IL-15 stimulation improved but did not fully preserve NK cell cytotoxicity under hypoxic conditions [12]. Hence, these data underscore the need for strategies that address the metabolic limitations imposed by the hypoxic TME directly.

Mitochondria are key regulators of cellular metabolism and immune cell fate [43]. They constantly undergo fission and fusion, reshaping their structure. Fission, mediated by DRP1 and FIS1, facilitates mitophagy, ROS production, apoptosis, and cell proliferation [44–49]. Fusion, controlled by MFN1, MFN2, and OPA1, forms tubular networks that enhance OXPHOS, increase Ca²^+^ flux, and protect cells from stress [50–54]. In hypoxic regions of human tumors, tumor-infiltrating NK cells exhibit increased DRP1 activation, driven by sustained mTOR signaling [8]. This results in mitochondrial fragmentation, reduced mitochondrial polarization, increased mtROS, and impaired cytotoxicity, which is corroborated by the findings of our study [8].

Given this background, and the profound changes in mitochondrial metabolism and content in hypoxic NK cells, we explored whether targeting DRP1 could protect NK cell function [8,23]. Our findings show that pharmacological DRP1 inhibition with Mdivi-1 restored mitochondrial defects and cytotoxicity. Similarly, Zheng *et al.* found that Mdivi-1 improved mitochondrial integrity and cytolytic activity of tumor-infiltrating NK cells exposed to hypoxia. However, due to concerns about the specificity and patient safety of Mdivi-1, we generated stable DRP1^KO^ NK cells using CRISPR-Cas9 technology. DRP1^KO^ NK cells proliferated normally in normoxia and maintained cytotoxic potential and mitochondrial content in hypoxia. To our knowledge, this is the first report describing the successful generation and functional evaluation of DRP1^KO^ NK cells.

Previous work in neuronal cells showed that CRISPR-Cas9-mediated KO of *DRP1* protected from ferroptotic cell death by preserving mitochondrial morphology and membrane potential, and stabilizing redox balance [55]. In addition, pharmacological or genetic inhibition of DRP1 can exert anti-apoptotic effects, for example by blocking Bax-dependent cytochrome c release [56,57]. While we observed similarly preserved mitochondrial content and membrane potential in DRP1^KO^ NK cells exposed to hypoxia, mtROS remained elevated and pro-apoptotic BAX expression was not fully suppressed, suggesting a redox impact and only partial protection against apoptotic signaling. In murine T cells, *Drp1* deletion has been reported to skew T cells toward a memory-like phenotype, at the expense of migration and cytoskeletal dynamics [58,59]. The precise mechanisms by which DRP1^KO^ NK cells maintain cytotoxicity in hypoxia are not yet fully understood and may involve a combination of altered mitochondrial ultrastructure, fusion-fission balance, and changes in metabolic pathways.

Despite functional differences, DRP1^KO^ and DRP1^WT^ NK cells displayed similar transcriptional profiles in hypoxia. This may, in part, reflect the shorter hypoxia exposure (24 h) used for RNA-seq, chosen to ensure high-quality transcriptomic data, in contrast to the 48 h exposure used in functional assays. Possibly, early-stage transcriptional changes do not fully reflect the later phenotypic divergence, consistent with the mitochondrial occupancy kinetics after 24 h in hypoxia. Moreover, as cells were not pre-activated with cytokines or target cells prior to transcriptomic or SCENITH™ profiling, the resulting data likely represent a more quiescent or basal state, potentially obscuring activation-dependent differences. One notable transcriptional difference was the behavior of mTORC1 signaling. While this pathway was downregulated in hypoxic DRP1^WT^ cells, it remained largely unchanged in DRP1^KO^ cells. Cytokine stimulation of NK cells results in mTOR activation, which is required for metabolic and functional responses, such as enhanced glycolysis and IFN-y production [36,60,61]. In many cell types, hypoxia dampens mTORC1 signaling, often involving stress-responsive pathways such as REDD1/TSC or AMPK [62–64]. While our transcriptomic data align with this general pattern, it contrasts Zheng *et al*., who reported increased mTORC1 activation in NK cells under hypoxic conditions [8]. Several factors could account for this discrepancy, including differences in cell type (primary NK cells vs NK-92 cells), hypoxia duration, culture conditions including cytokines, and the specific readouts used to assess mTORC1 activity. Although RNA-seq provides only an indirect view of mTORC1 signaling, established mTORC1-responsive transcriptional programs are observed in cells with high mTORC1 activity [65]. In this context, the preserved mTORC1-associated transcriptional output we observe under hypoxia may contribute to the maintained functionality of NK cells. Future studies incorporating direct biochemical measurements will be required to clarify the precise role of mTORC1 in hypoxic NK cells.

Oxygen availability in solid tumors is highly heterogeneous, ranging from near-anoxic conditions (<0.1% O₂) within necrotic cores to milder hypoxia (up to ∼5% O₂) towards invasive edges. We used chronic 1% O₂ to investigate the detrimental effect of strong and persistent tumor hypoxia on NK cell mitochondrial biology and effector functioning. Since hypoxia exposure *in vitro* is static, we included PDAC patient-derived 3D microtumors to approximate spatial oxygen gradients and architectural complexity. The transcriptional heterogeneity between the patient-derived organoids strongly predicted the CAF response to CAR NK cells in the microtumor models. Patient 1 exhibited enrichment for TGF-β/EMT and epithelial-hypoxic programs, characteristic of immune-excluding PDAC states. TGF-β signaling is a central driver of myCAF differentiation, extracellular matrix deposition, and direct NK cell suppression [66,67], providing a mechanistic rationale for the complete resistance observed. Patient 2 organoids displayed proliferative and apoptotic stress programs, a transcriptional state associated with increased expression of stress ligands and enhanced susceptibility to granzyme-mediated cytotoxicity [68], putatively resulting in a more permissive context for CAR NK cell cytotoxicity. Patient 3 organoids showed strong glycolytic and hypoxic signatures, transcriptional states known to create metabolically suppressive microenvironments that impair immune effector cell mitochondrial function through lactate accumulation, acidosis and nutrient competition [69,70]. These conditions selectively disabled DRP1^WT^ NK cells, which are sensitive to hypoxia-induced mitochondrial fragmentation [8], but were overcome by DRP1^KO^ NK cells, which maintain mitochondrial fitness under hypoxic stress. While these associations rely on organoid transcriptional data, it supports the importance of mitochondrial dynamics and fitness of NK cells, which both dictate immune cell fate and function [23,71]. Together, these finding show that tumor-intrinsic programs play decisive roles in determining CAR NK cell efficacy, highlighting the importance of matching NK cell engineering strategies to patient-defined resistance states, for which large cohorts with diverse patients are warranted. Although patient-derived *in vitro* models allow to incorporate such patient heterogeneity, they lack dynamic oxygen cycling and full stromal diversity, including vascular influences and regulatory immune cells. Therefore, relevant *in vivo* models are ultimately required for cell therapy research with translational purposes. Overall, tumor oxygen constraints represent an additional challenge in designing cellular therapies that are adequately equipped to perform consistently across the heterogeneity within and between patient tumors.

In conclusion, our findings show that hypoxia exerts wide-ranging effects on NK cell metabolism and function, which cannot be fully rescued by cytokine-armed CARs. Our data also indicates that mitochondrial dynamics are central to this phenomenon, with disruption of DRP1 in NK cells preserving mitochondrial function and cytotoxic effector function in hypoxia. Such mitochondrial modulation might be required for effective functioning of CAR NK cells in solid cancers.

## Supplementary Material

250913681_Verhezen_Revision_SupplementaryData_clean.docx

## Data Availability

The datasets used and analyzed during the current study are available from the corresponding author on reasonable request. Data was also included in the preprint of this article on BioRxiv: Verhezen T, Van den Eynde A, et al. DRP1 depletion protects NK cells against hypoxia-induced dysfunction. BioRxiv. 2025 June 30. doi: 10.1101/2025.06.23.661011
