# Supplementary material for: DRP1 depletion protects NK cells from hypoxia-induced dysfunction
Source: Redox Rep. 2026 Feb 18;31(1):2626181. doi: 10.1080/13510002.2026.2626181 (PMC12918307; doi:10.1080/13510002.2026.2626181)
Supplement: 250913681_Verhezen_Revision_SupplementaryData_clean.docx [file YRER_A_2626181_SM8745.docx]

**DRP1 depletion protects NK cells from hypoxia-induced dysfunction**

Tias Verhezen^1^, Astrid Van den Eynde^1^, Peter Verstraelen^2^, Laura Gehrcken^1^, Gabriele Palmiotto^1,3^, Ho Wa Lau^1^, Winnok H. De Vos^2,4,5^, Sanne van der Heijden^1^, Louize Brants^1^, Jöran Melis^1^, Jonas Van Audenaerde^1^, Felicia Rodrigues Fortes^1^, Maxim Le Compte^1^, Geert Roeyen^6^, Hans Prenen^7^, Diana Campillo-Davo^8^, Eva Lion^8^, Rafael J Argüello^9^, Steven Van Laere^1^, Filip Lardon^1^, Christophe Deben^1^, An Wouters^1^, Evelien Smits^1^, Jorrit De Waele^1^

^1^ Center for Oncological Research (CORE), Integrated Precision and Personalized Oncology Network (IPPON), University of Antwerp, Belgium

^2^ Laboratory of Cell Biology and Histology, University of Antwerp, Belgium

^3^ School of Medicine and Surgery, University of Bologna, Italy

^4^ Antwerp Centre for Advanced Microscopy, University of Antwerp, Belgium

^5^ µNEURO Research Centre of Excellence, University of Antwerp, Belgium

^6^ Department of Hepatobiliary Transplantation and Endocrine Surgery, Antwerp University Hospital, Belgium

^7^ Department of Oncology, Multidisciplinary Oncology Center Antwerp, Antwerp University Hospital, Belgium

^8^ Laboratory of Experimental Hematology (LEH), Vaccine and Infectious Disease Institute (VAXINFECTIO), Faculty of Medicine and Health Sciences, University of Antwerp, Edegem, Belgium

^9^ Aix Marseille University, CNRS, INSERM, CIML, Centre d'Immunologie de Marseille-Luminy, France

**Supplementary data**

**Supplementary Tables**


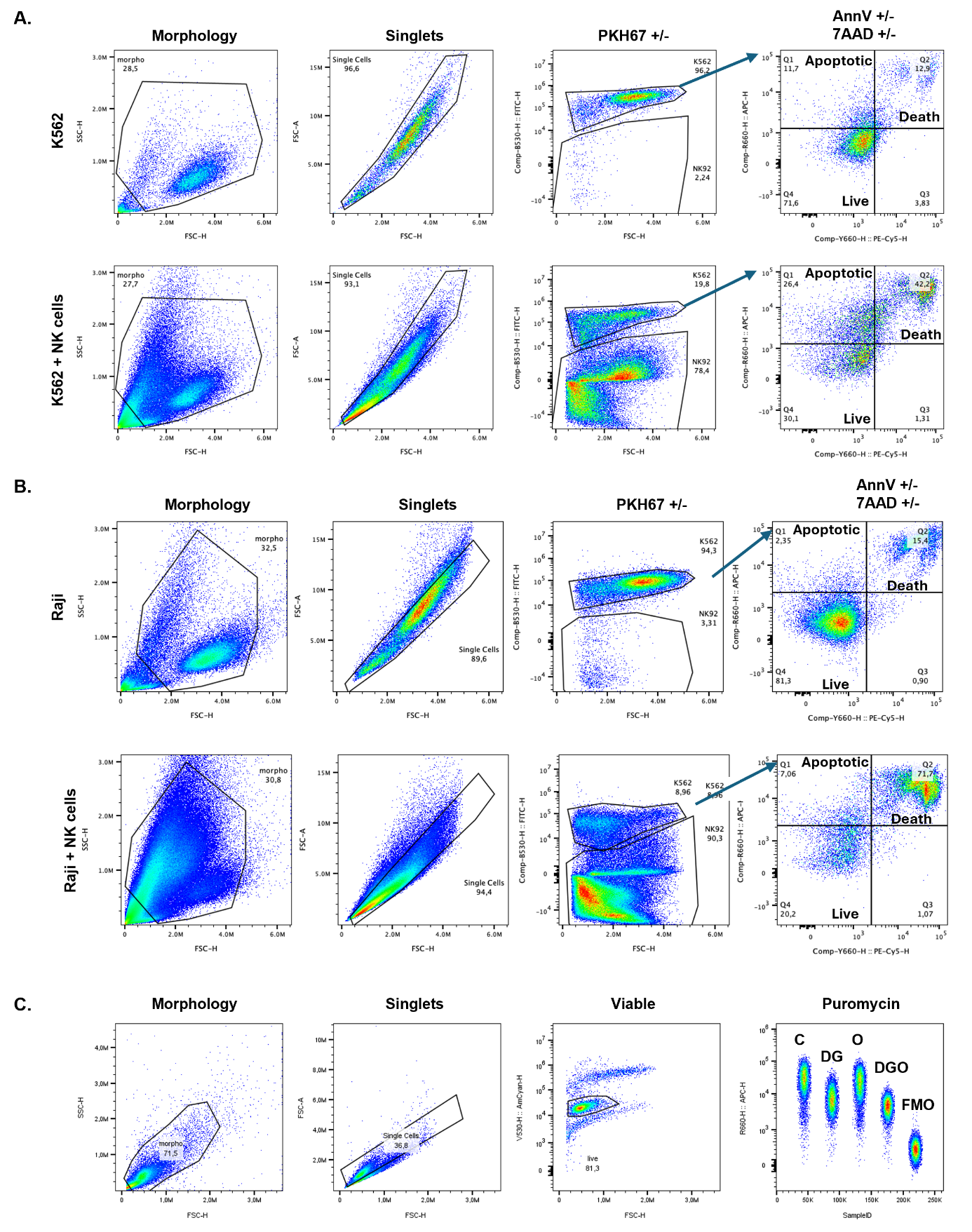


**Supplementary Figure S1. Gating strategies for flow cytometric cytotoxicity and SCENITH assays.** A-B. Gating strategy for co-culture assays with K562 (A) or Raji (B) cells and NK cells on flow cytometry; C. Gating strategy for the metabolic SCENITH assay quantifying ATP consumption via puromycin incorporation to analyze protein translation. Cells were treated with control (Co), 2-deoxyglucose (DG), oligomycin (O), or the combination (DGO). FMO, fluorescence-minus-one control.


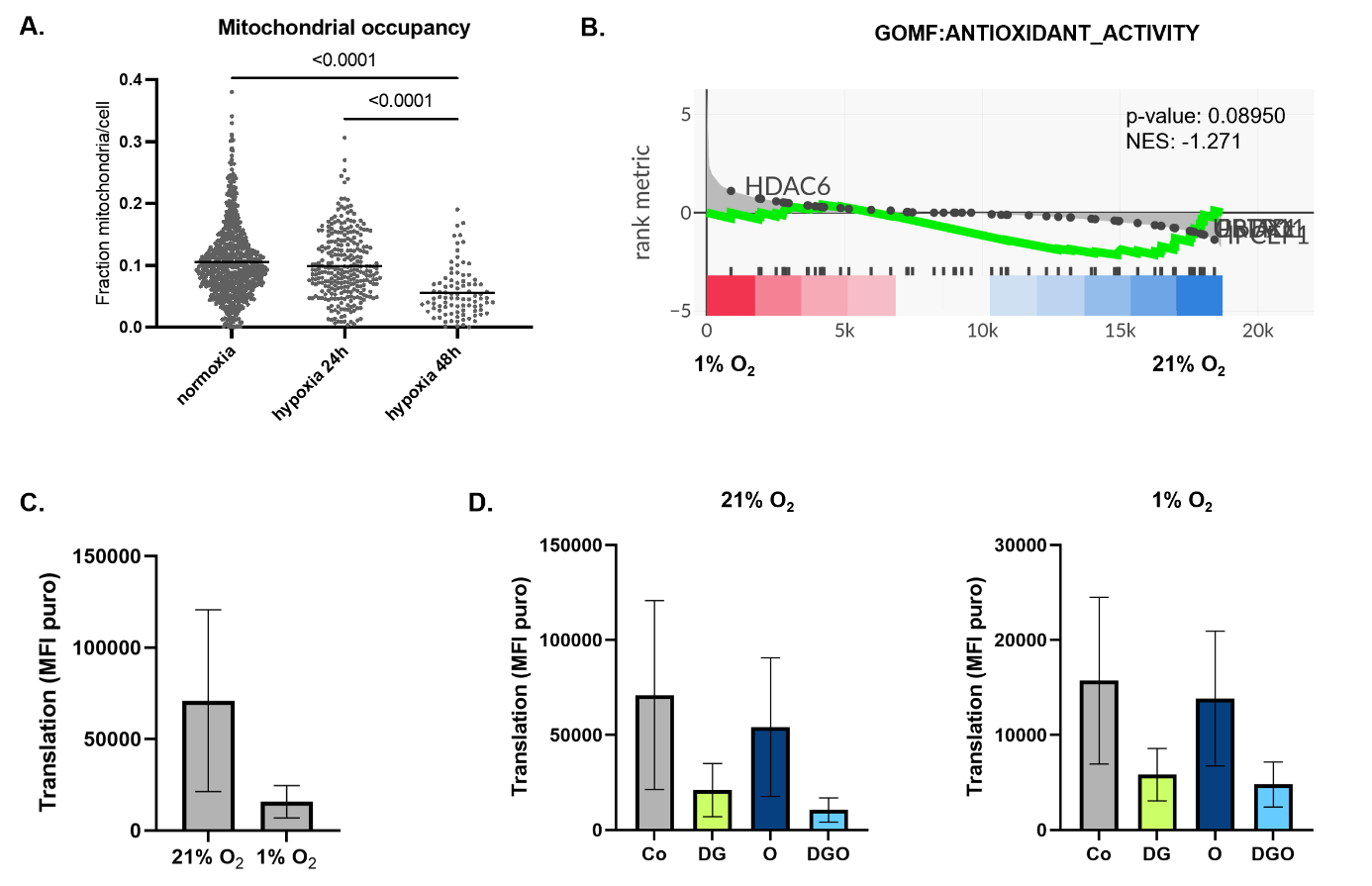


**Supplementary Figure S2. Effect of hypoxia on mitochondrial features of NK cells.** A. Mitochondrial occupancy over time in hypoxic culturing, determined by confocal microscopy; B. Transcriptomic signature for antioxidant activity in wild-type NK cells in hypoxia versus normoxia; C-D. Assessment of protein translation in NK cells cultured in normoxia or hypoxia for 48 hours, as measured by puromycin incorporation (SCENITH assay). Cells were treated with control (Co), 2-deoxyglucose (DG), oligomycin (O), or the combination (DGO).


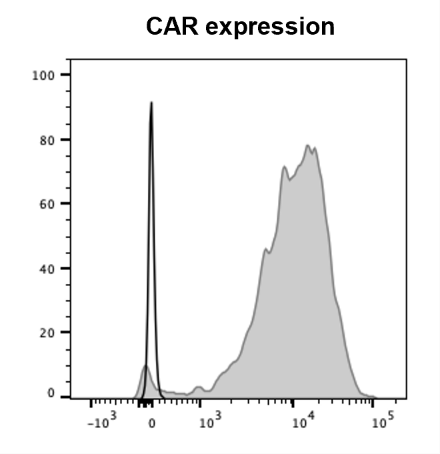


**Supplementary Figure S3.** **CAR expression of wild-type NK cells.** Representative histograms of CAR expression on CAR NK cells determined by flow cytometry.


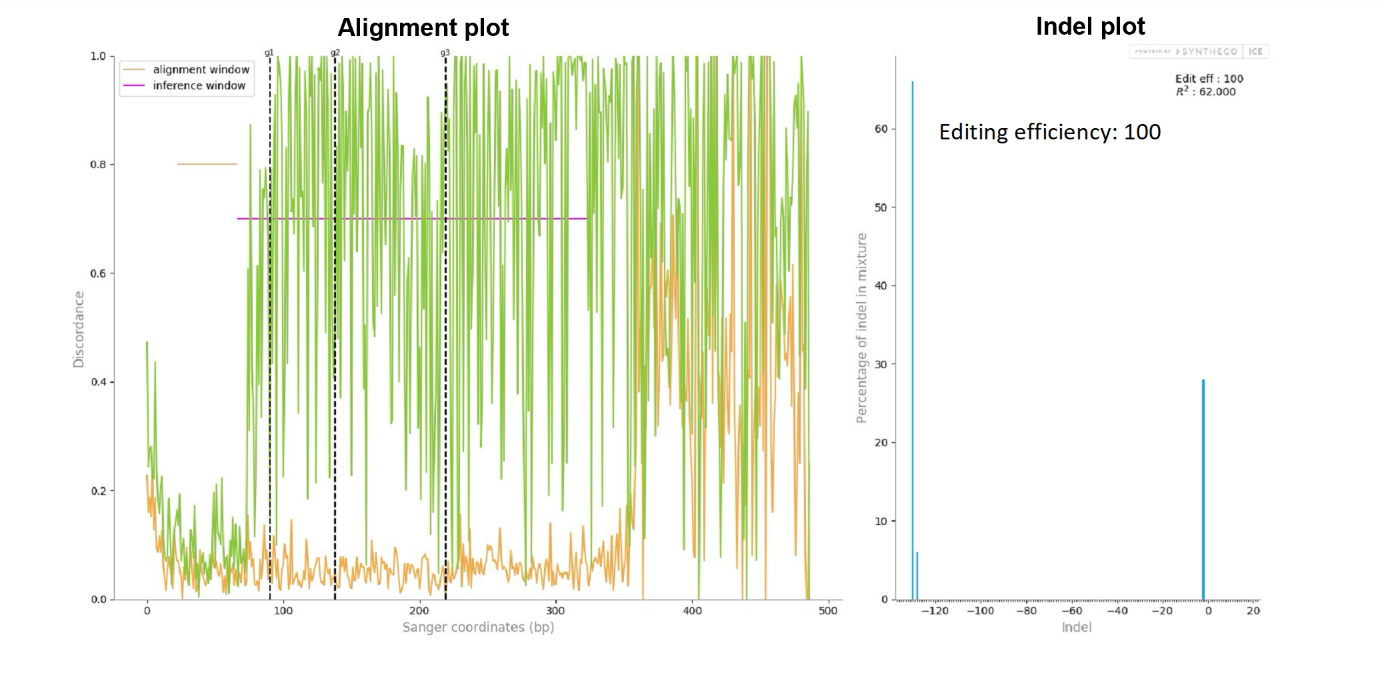


**Supplementary Figure S4.** **Analysis of Editing Efficiency of the DRP1 gene in NK cells.** Determined by Sanger sequencing and ICE analysis. (left) An alignment plot displaying the aligned control (orange) and edited (green) sequences; (right) an indel plot depicting the anticipated range of insertions and deletions within the edited gene locus and the editing efficiency.


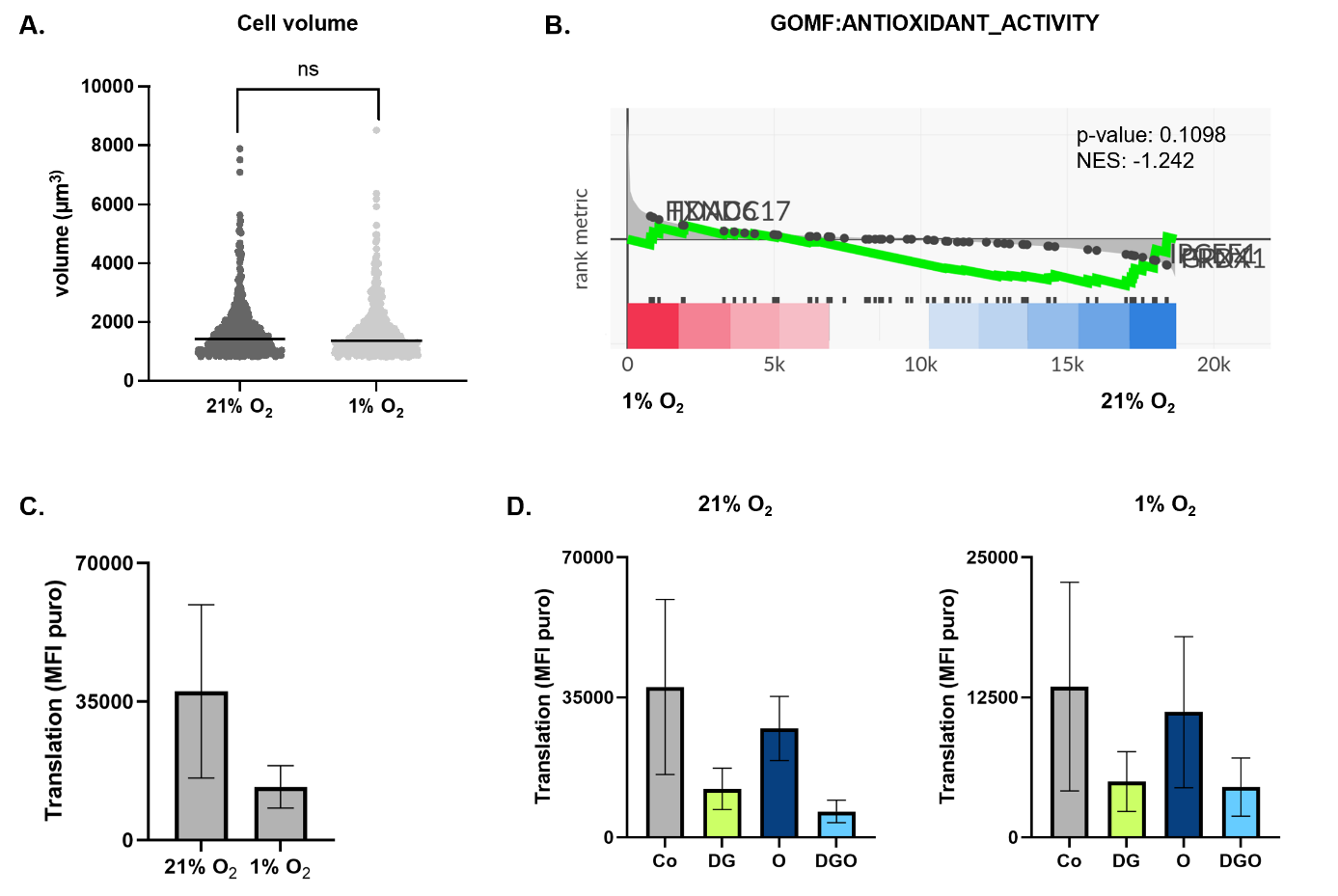


**Supplementary Figure S5.** **Metabolic features of DRP1^KO^ NK cells.** A. Cell volume determined by confocal microscopy; B. Transcriptomic signature for antioxidant activity in DRP1^KO^ NK cells in hypoxia versus normoxia; C-D. Assessment of protein translation in NK cells cultured in normoxia or hypoxia for 48 hours, as measured by puromycin incorporation (SCENITH™ assay). Cells were treated with control (Co), 2-deoxyglucose (DG), oligomycin (O), or the combination (DGO).


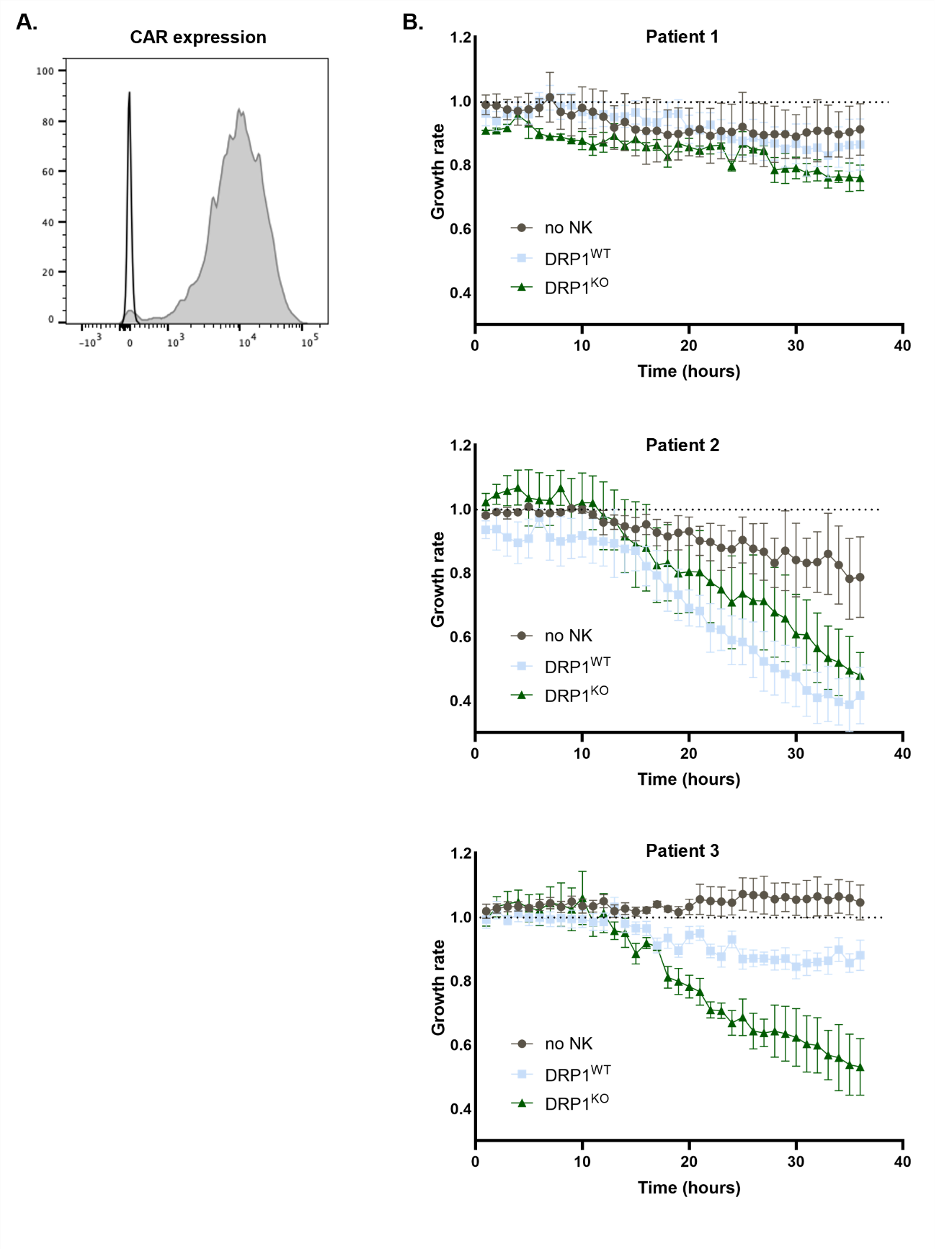


**Supplementary Figure S6. CAR expression of DRP1^KO^ cells and longitudinal microtumor data.** A. Representative histograms of CAR expression on DRP1^KO^ CAR NK cells determined by flow cytometry. B. Longitudinal growth of microtumors was monitored over 36 hours, based on the fluorescent signal of CD70^+^ CAFs; growth curves over time for Patients 1-3.

**Supplementary Tables**

**Table S1. TCGA cancer type abbreviations and corresponding full names (ordered as in Figure 2A).**

| **TCGA Code** | **Full Cancer Type Name** |
| --- | --- |
| ACC | Adrenocortical carcinoma |
| BRCA | Breast invasive carcinoma |
| PAAD | Pancreatic adenocarcinoma |
| SARC | Sarcoma |
| LIHC | Liver hepatocellular carcinoma |
| CHOL | Cholangiocarcinoma |
| ESCA | Esophageal carcinoma |
| PCPG | Pheochromocytoma and Paraganglioma |
| KIRP | Kidney renal papillary cell carcinoma |
| STAD | Stomach adenocarcinoma |
| KICH | Kidney chromophobe |
| TGCT | Testicular germ cell tumors |
| THYM | Thymoma |
| BLCA | Bladder urothelial carcinoma |
| PRAD | Prostate adenocarcinoma |
| THCA | Thyroid carcinoma |
| MESO | Mesothelioma |
| LGG | Brain lower grade glioma |
| COADREAD | Colon and rectum adenocarcinoma |
| GBM | Glioblastoma multiforme |
| DLBC | Lymphoid neoplasm diffuse large B-cell lymphoma |
| UCEC | Uterine corpus endometrial carcinoma |
| SKCM | Skin cutaneous melanoma |
| LAML | Acute myeloid leukemia |
| HNSC | Head and neck squamous cell carcinoma |
| LUAD | Lung adenocarcinoma |
| OV | Ovarian serous cystadenocarcinoma |
| KIRC | Kidney renal clear cell carcinoma |
| LUSC | Lung squamous cell carcinoma |
| UCS | Uterine carcinosarcoma |
| CESC | Cervical squamous cell carcinoma and endocervical adenocarcinoma |
| UVM | Uveal melanoma |
